# Human herpes virus 8 replication during disseminated tuberculosis in a man with human immunodeficiency virus: a case report

**DOI:** 10.1186/1752-1947-3-113

**Published:** 2009-11-09

**Authors:** Sarra Inoubli, Laurence Toutous-Trellu, Gieri Cathomas, Eric Oksenhendler, Bernard Hirschel, Emmanuelle Boffi El Amari

**Affiliations:** 1 Département des maladies infectieuses, Hôpital Cantonal Universitaire de Genève, Genève, Switzerland; 2 Kantonales Institute für Pathologie Liestal, Liestal, Switzerland; 3 Département d'Immunologie Clinique, Hôpital Saint-Louis, Paris, France

## Abstract

**Introduction:**

Human herpes virus 8 (HHV-8) is mainly responsible for the development of Kaposi's sarcoma and multicentric Castleman's disease in immunocompromised patients with untreated human immunodeficiency virus. Positive viral loads have been described in cases of Kaposi's sarcoma and multicentric Castleman's disease, with higher values found in the latter. We describe the case of a patient with HIV in whom a high level of HHV-8 replication was detected and who contracted an opportunistic disease other than multicentric Castleman's disease or Kaposi's sarcoma.

**Case presentation:**

A 25-year-old man of West African origin with HIV complained of asthenia, weight loss, fever, and abdominal pain. Physical examination revealed that the patient had adenopathies and hepatosplenomegaly, but no skin or mucosal lesions were seen. Our first presumptive diagnosis was disseminated tuberculosis. However, since the cultures (sputum, bronchoalveolar lavage, blood, urine and lymph node biopsies) for mycobacteria were negative, the diagnosis was expanded to include multicentric Castleman's disease which was supported by high HHV-8 viral loads in the patient's blood: 196,000 copies/ml in whole blood, 39,400 copies/ml in plasma and 260 copies/10E5 in peripheral blood mononuclear cells. However, the histology and positive polymerase chain reaction assay for *Mycobacterium tuberculosis *complex of a second lymph node biopsy enabled us to conclude that the patient had disseminated tuberculosis and we started the patient on antituberculosis treatment.

We analyzed the HHV-8 deoxyribonucleic acid in two other plasma samples (one from six months earlier and the other was 10 days after the positive test) and both yielded negative results. A search for latent and lytic HHV-8 antibodies confirmed that the patient was seropositive for HHV-8 before this episode.

**Conclusion:**

We describe the case of a patient with HIV who tested positive for asymptomatic HHV-8 replication during an opportunistic disease suggestive of multicentric Castleman's disease. The initial analysis was nullified by the diagnosis of a disease that was unrelated to HHV-8. This case report underlines the need to clarify the full clinical meaning and implication of a positive HHV-8 viral load in patients with AIDS. The diagnosis of multicentric Castleman's disease needs to be studied further to determine its sensitivity and specificity. Finally, when faced with the dilemma of urgently starting chemotherapy on a patient whose condition is deteriorating and whose clinical presentation suggests multicentric Castleman's disease, high HHV-8 viral loads should be interpreted with caution and histological analysis of lymph nodes or liver biopsies should be obtained first.

## Introduction

Human herpes virus 8 (HHV-8) is associated with the development of Kaposi's sarcoma (KS) and multicentric Castleman's disease (MCD) mostly in immunocompromised patients with untreated human immunodeficiency virus (HIV). MCD is an atypical lymphoproliferative disease characterized by systemic symptoms that include fever, weakness, severe weight loss, generalized lymphadenopathy and hepatosplenomegaly. The pathological examination of lymph nodes reveals angiofollicular hyperplasia, atrophic germinal centers surrounded by concentric layers of small B cells with a typical onion skin feature, and intense interfollicular plasma cell hyperplasia. Immunohistochemistry using antibodies against latent nuclear antigen of HHV-8 allows the detection of B cells infected with HHV-8. These cells have undergone plasma cell differentiation and are mainly located in the mantle zone. This method of staining is particularly useful when typical features such as onion skin lesions are lacking and when intense interfollicular hyperplasia may be considered as non-specific or secondary to HIV infection. MCD is clinically very aggressive and can progress to frank monoclonal lymphoma. The median survival is 14 to 48 months from the time of diagnosis [1,2].

In addition to antiretroviral treatments, patients with HIV are treated for MCD with chemotherapy using etoposide, vinblastine, and anti-CD20 (rituximab) or combined chemotherapy (CHOP), which is associated with major side effects [3].

The clinical presentation of MCD resembles an opportunistic infection where chemotherapy would be strongly contraindicated. It is therefore crucial to establish a rapid and correct diagnosis. HHV-8 deoxyribonucleic acid (DNA) can be detected in the blood by gene amplification. Positive values are found during KS and MCD, but levels rise in magnitude during active MCD [3-5].

We describe the case of a patient with HIV infection and severe constitutional symptoms. An extensive search for an opportunistic disease was initially negative. However, just before the patient was initiated on chemotherapy for presumed MCD, high levels of HHV-8 DNA were detected. A repeat lymph node biopsy finally established the diagnosis of tuberculosis. For a description of the methods, please see Additional file 1.

## Case presentation

A 25-year-old man of West African origin was diagnosed with HIV in August 2006. In January 2007, the patient started complaining about multiple bouts of asthenia, weight loss, fever, and abdominal pain. He was hospitalized in February 2007. On physical examination, he had fever (38.9°C), multiple inguinal and axillary adenopathies, and hepatosplenomegaly. His chest examination was clear, and no skin or mucosal lesions were seen.

The patient's blood count revealed a haemoglobin level of 113 g/l and a low platelet count of 70 g/l. There were mild liver test disturbances (aspartate aminotransferase 103 U/l (normal range: 14-50 U/l), alanine aminotransferase 69 U/l (normal range: 12-50 U/l), alkaline phosphatase 350 U/L (normal range: 30-125 U/l), gamma-glutamyltransferase 271 umol/l (normal range: 9-40 U/l) and lactate dehydrogenase 529 U/l (normal range: 125-240 U/l). His CD4+ T-cell count was 224 cells/mm^3 ^(13%) and his HIV viremia was 2.7E6 copies/ml (Table 1).

**Table 1:** Laboratory values

	Patient values				
	
	Normal range	Aug 06	Oct 06	Feb 07	Mar 07
Lymphocyte count, g/l	1-4.5	1.99	2.06	2.75	

T-cell count cell/mm^3^	1050-3500	1840	2062	1722	

CD4 count cell/mm^3^	600-1950	386	433	224	280

CD8 count cell/mm^3^	300-1100	957	1093	1050	

HIV viral load copies/ml	<40-10E7	10E5	5.7E5	2.7E6	2.0E6

HHV-8 whole blood copies/ml		neg		196 000	neg

HHV-8 plasma copies/ml		neg		39 400	neg

HHV-8 PBMC copies/10E5 cells		neg		260	neg

HHV-8 serology latent (title)	1/512		1/512	1/512	

HHV-8 serology lytic (title)	1/1024		1/1024	1/1024	

HHV-8, human herpesvirus 8; HIV, human immunodeficiency virus; PBMC, peripheral blood mononuclear cells

A computed tomography scan revealed multiple mediastinal, retroperitoneal and pelvic lymphadenopathies, hepatosplenomegaly, and disseminated pulmonary micronodules. A tuberculin skin test and whole blood interferon gamma assay were both positive.

Cultures for mycobacteria in sputum, bronchoalveolar lavage fluid, urine and blood were performed, but no acid-fast bacilli were seen. An axillary lymph node biopsy showed non-specific reactive lymphoid hyperplasia. A polymerase chain reaction (PCR) search revealed neither the presence of *Mycobacterium tuberculosis *complex DNA nor clonal B or T cells.

At this point, due to the absence of mycobacteria in all samples and because the patient's presentation was also compatible with MCD, his HHV-8 DNA was measured. The results revealed an HHV-8 viral load of 198,000 copies/ml in whole blood, 260 copies/10E5 cells in peripheral blood mononuclear cells (PBMCs), and 39,400 copies/ml in plasma. Physical examination did not reveal any mucocutaneous signs of KS.

The patient underwent a second lymph node biopsy, which revealed areas of necrosis surrounded by giant cell granulomas. The results of Ziehl-Neelson staining were again negative, but the PCR search for *Mycobacterium tuberculosis *complex was positive, which enabled us to conclude that the patient had disseminated tuberculosis. There was no evidence for MCD or any other lymphoproliferative processes.

The patient was then started on isoniazid, rifampicin, ethambuthol and pyrazinamide treatments, and his condition subsequently improved. The patient was started on highly active antiretroviral therapy (HAART) two months later and once the antituberculosis treatment had been simplified. Eventually, *Mycobacterium tuberculosis *grew in the cultures of the first bronchial aspirate and then in the second biopsy.

We analyzed the HHV-8 DNA in two other plasma samples: one dating back to August 2006, and one taken 10 days after the positive sample. In between these two periods, the patient had received methyprednisolone 250 mg three times daily for two days. Both of the samples were negative. All the samples were double-checked in the same laboratory. Genotypic analyses of HIV in both the positive and negative samples were identical. A search for latent and lytic HHV-8 antibodies was performed on the August 2006 sample. It confirmed that the patient was HHV-8 seropositive before this episode. No variation in the titles between August 2006 and March 2007 was noted.

## Discussion

The clinical presentation of fever, weight loss, generalized lymphadenopathy and enlargement of the liver and spleen in our patient was suggestive of tuberculosis. However, as the search for mycobacteria was initially negative, the diagnosis was expanded to include MCD. This was supported by the clinical presentation and the high HHV-8 viral load measured in the patient's blood plasma. Finally, the histology and positive PCR assay of the second lymph node biopsy firmly established the diagnosis of disseminated tuberculosis. This was further supported by the fact that the patient was apparently cured by antituberculosis treatment. This evolution, as well as two lymph node biopsies without evidence of MCD made it unlikely that the patient had both tuberculosis and MCD. Physical examination never revealed any signs of KS.

This case reveals that high levels of HHV-8 can be measured during an opportunistic infection other than KS or MCD, suggesting that HHV-8 infection can be transiently reactivated in an apparently asymptomatic way.

HHV-8 DNA can be detected in whole blood, plasma and, most frequently, in PBMCs. Positive values in PBMCs have been found in up to 73.2% of reported patients with KS [6] and in all patients with MCD or primary effusion lymphomas [7]. Oksenhendler *et al*., in a prospective study of 23 patients with HIV and MCD, measured a mean value of 4,77 log copies/ug DNA in the PBMCs of all the patients under study. This value was slightly higher in patients with MCD and KS. It is also important to note that two of the 12 patients with asymptomatic HHV-8 infection had low detectable levels of 2,91 log copies [5]. In a retrospective study involving eight patients with HIV and with either KS or KS and MCD, Boivin *et al*. measured HHV-8 DNA levels of up to as high as 47,210/10E5 cells in PBMCs and 256/10 ul (25,600 cells/ml) in plasma in the patients with MCD. The study also showed that most of the patients with KS had undetectable values or, at most, 135 copies/10E5 cells [4].

HHV-8 DNA detection predicts the clinical evolution and the response to treatment of both KS and MCD, but its positive predictive value is still unknown [4,5]. On the contrary, it must be noted that the negative predictive value of HHV-8 DNA in PBMCs, when associated with the clinical and biological symptoms of MCD, is quite high. In our patient, the high viral concentration found in his plasma may suggest the replication and non-specific activation of a latent HHV-8 infection in the context of inflammatory dysregulation associated with another infection, instead of a reactivation associated with MCD or KS [8].

There are few published examples of HHV-8 reactivation without evidence of MCD or KS. Van der Kuyl *et al*. detected HHV-8 DNA in the PBMCs of 14% of reported cases of asymptomatic HHV-8 infection (median 2, 0 log/10E6 cells). Half of the patients with HHV-8 reactivation had concomitant cytomegalovirus (CMV) infections [9]. Lisco *et al*. detected a positive HHV-8 viral load in the PBMCs of pregnant HIV-infected women during their second and third trimesters, which suggest that pregnancy may induce HHV-8 replication [10]. Finally, Hudnall *et al*. detected active HHV-8 replication in healthy HHV-8 seropositive renal transplant patients [11].

*In vitro *studies showed that inflammatory cytokines, such as interferon gamma, induce HHV-8 replication in the PMBCs of HIV-infected patients and in primary effusion lymphoma cell lines [8,12,13]. This experimental evidence tends to imply that increased plasmatic levels of HHV-8 are not exclusively indicative of the presence of MCD or KS, but could simply reflect inflammatory dysregulation associated with HIV infection, for example.

In non-HIV immunosuppressed patients such as transplant recipients, other herpes viruses such as CMV or Epstein-Barr virus are known to reactivate. A regular follow-up of the DNA load is thus suggested to detect continuous replication and elevated values in order to prevent the development of the disease and to adapt the immunosuppressive treatment [14].

## Conclusion

This case report underlines the need to clarify the quantification as well as the full clinical meaning and implication of a positive HHV-8 viral load in patients with HIV. The potential for diagnosing MCD needs to be studied further to determine its sensitivity and specificity. Finally, when faced with the dilemma of urgently starting chemotherapy in a patient whose condition is deteriorating and whose clinical presentation suggests MCD, high whole blood and plasmatic HHV-8 viral loads should be interpreted with caution and histological analysis of lymph nodes should be obtained first in order to establish the definitive diagnosis of MCD.

## Abbreviations

CMV: cytomegalovirus; DNA: deoxyribonucleic acid; HAART: highly active antiretroviral therapy; HHV-8: human herpesvirus 8; HIV: human immunodeficiency virus; KS: Kaposi's sarcoma; MCD: multicentric Castleman's disease; PBMC: peripheral blood mononuclear cells; PCR: polymerase chain reaction

## Consent

Written informed consent was obtained from the patient for publication of this case report and any accompanying images. A copy of the written consent is available for review by the Editor-in-Chief of this journal.

## Competing interests

The authors declare that they have no competing interests.

## Authors' contributions

SI and EB were the major contributors in writing the manuscript. EO analyzed and interpreted the patient's data regarding the HHV-8 infection and strong suspicion of MCD. GC performed the histological examination of the adenopathies and measured the HHV-8 levels. BH supervised the HIV and tuberculosis infection. TL was the referent dermatologist for this case. All authors read and approved the final manuscript.

## Supplementary Material

Additional file 1 **Methods**. The data provided represent the methods employed in the detection and quantification of HHV-8 viral load in the plasma, whole blood and PBMC. Click here for file
