# Supplementary material for: Human herpes virus 8 replication during disseminated tuberculosis in a man with human immunodeficiency virus: a case report
Source: J Med Case Rep. 2009 Nov 9;3:113. doi: 10.1186/1752-1947-3-113 (PMC2783054; doi:10.1186/1752-1947-3-113)
Supplement: Additional file 1 — Methods. The data provided represent the methods employed in the detection and quantification of HHV-8 viral load in the plasma, whole blood and PBMC. [file 1752-1947-3-113-S1.doc]

**Additional file 1**

**Methods**

The detection and quantification of HHV-8 viral load in the plasma, whole blood and PBMC were done by qualitative and quantitative PCR. DNA was extracted using an EZ1-DNA extraction robot (Qiagen, Hombrechtikon, Switzerland) according to the manufacturer’s instructions. For qualitative evaluation, a nested PCR for the HHV-8 ORF 26 region was amplified as previously described [1]. All reactions were performed in duplicates. For the calculation of molecule quantity, serial dilutions of ORF 26 containing HHV-8 plasmid DNA were analyzed and a standard curve was established as previously described [2]. Results were expressed in copies/ml for whole blood and plasma and in copies per 100,000 cells for PBMC.

**References**

1. Cathomas G, Stalder A, McGandy CE, Mihatsch MJ: **Distribution of human herpesvirus 8 DNA in tumorous and non-tumorous tissue of patients with acquired immunodeficiency syndrome with and without Kaposi's sarcoma.** *Mod Pathol* 1998, **11:**415-420.

2. Zsikla V, Hailemariam S, Baumann M, Mund MT, Schaub N, Meier R, Cathomas G: **Increased rate of Helicobacter pylori infection detected by PCR in biopsies with chronic gastritis.** *Am J Surg Pathol* 2006, **30:**242-248.
